# Fluid Administration in Emergency Room Limited by Lung Ultrasound in Patients with Sepsis: Protocol for a Prospective Phase II Multicenter Randomized Controlled Trial

**DOI:** 10.2196/15997

**Published:** 2020-08-26

**Authors:** Mouhand F H Mohamed, Nathalie M Malewicz, Hanan Ibrahim Zehry, Dina A Monim Hussain, Judah Leão Barouh, Adriana V Cançado, Jeancarllo Sousa Silva, Salah Suwileh, Jose Retamal Carvajal

**Affiliations:** 1 Department of General Internal Medicine Hamad Medical Corporation Doha Qatar; 2 Department of Anesthesiology Yale University School of Medicine New Haven, CT United States; 3 Intensive Care Medicine and Pain Management Department of Anaesthesiology Medical Faculty of Ruhr-University Bochum Germany; 4 Department of Physical Medicine and Rehabilitation Spaulding Rehabilitation Hospital and Massachusetts General Hospital Harvard Medical School Boston, MA United States; 5 Graduate School of Biomedical and Health Sciences Hiroshima University Hiroshima Japan; 6 Faculty of Nursing, Suez Canal University Ismailia Egypt; 7 Department of Obstetrics and Gynecology Hamad Medical Corporation Doha Qatar; 8 Radiology Department Santa Casa Belo Horizonte Belo Horizonte Brazil; 9 Division of Oncological Surgery State University of Amazonas Manaus Brazil; 10 Division of Oncological Surgery Getulio Vargas University Hospital Amazonas Brazil; 11 Faculty Of Emergency Medicine Universidad Del Desarrollo-Clinica Alemana De Santiago Santiago Chile

**Keywords:** sepsis, fluid resuscitation, PaO2/FiO2, B-Lines, point-of-care ultrasound, pulmonary edema, oxygenation, outcomes, emergency department, ultrasound, lung

## Abstract

**Background:**

Sepsis remains a major health challenge with high mortality. Adequate volume administration is fundamental for a successful outcome. However, individual fluid needs differ between patients due to varying degrees of systemic vasodilation, circulatory flow maldistribution, and increased vascular permeability. The current fluid resuscitation practice has been questioned. Fluid overload is associated with higher mortality in sepsis. A sign of fluid overload is extravascular lung water, seen as B lines in lung ultrasound. B lines correlate inversely with oxygenation (measured by a ratio of the partial pressure of arterial oxygen to the fraction of inspired oxygen ie, PaO2/FiO2). Thus, B lines seen by bedside ultrasound may have a role in guiding fluid therapy.

**Objective:**

We aim to evaluate if fluid administration guided by lung ultrasound in patients with sepsis in emergency departments will lead to better oxygenation and patient outcomes than those in the standard therapy.

**Methods:**

A phase II, multicenter, randomized, open-label, parallel-group, superiority trial will be performed. Patients will be recruited at emergency departments of the participating centers. A total of 340 patients will be randomly allocated to the intervention or standard-of-care group (30mL/kg). The intervention group will receive ultrasound-guided intravenous fluid until 3 B lines appear. The primary outcome will be oxygenation (measured as PaO2/FiO2 ratio) at 48 hours after starting intravenous fluid administration. Secondary outcomes will be patients’ outcome parameters, including oxygenation after 15 mL/kg fluid at 6, 12, 24, and 48 hours; sepsis progress through Sequential Organ Failure Assessment (SOFA) scores; pulmonary edema evaluation; and 30-day mortality.

**Results:**

The trial will be conducted in accordance with the Declaration of Helsinki. Institutional review board approval will be sought after the participating sites are selected. The protocol will be registered once the institutional review board approval is granted. The trial duration is expected to be 1.5-2.5 years. The study is planned to be performed from 2021 to 2022, with enrollment starting in 2021. First results are expected in 2022. Informed written consent will be obtained before the patient’s enrollment in the study. An interim analysis and data monitoring will ensure the patient safety. The results will be published in a peer-reviewed journal and discussed at international conferences.

**Conclusions:**

This is a protocol for a randomized control trial that aims to evaluate the role of bedside ultrasound in guiding fluid therapy in patients with sepsis via B lines evaluation.

**International Registered Report Identifier (IRRID):**

PRR1-10.2196/15997

## Introduction

Sepsis is a significant cause of in-hospital mortality [1]. Prompt and adequate intravenous (IV) fluid therapy is essential in the treatment of sepsis and to reduce sepsis mortality [2,3]. Especially, immediate initial resuscitation in an emergency department can impact patients’ outcomes [4-6]. In 2001, fluid resuscitation with the volume of 30 mL/kg, as early-goal directed-therapy, was added to the standard therapy for sepsis in the emergency department [7]. However, later studies on the Protocolized Care for Early Septic Shock (ProCESS), Australasian Resuscitation in Sepsis Evaluation (ARISE), and Protocolised Management in Sepsis (ProMISe) debated this initial fluid amount. These studies suggested that the fixed amount approach is not appropriate and beneficial for all patients and can be even harmful [8-11]. Even the 2016 definitions of sepsis and septic shock (Sepsis-3) failed to redefine this amount [12-14].

Studies suggest that only 50% of patients with sepsis respond positively to increased fluid administration [15] since the mechanism of circulatory compromise in sepsis is not related to actual hypovolemia. Therefore, excessive volumes are considered harmful and can cause myocardial dysfunction, pulmonary congestion, and decreased cardiac output [16].

Techniques like passive leg raising and inferior vena cava monitoring are useful to identify additional fluid responsiveness in patients [17-19]. However, these tests are cumbersome and time-consuming, especially in the busy emergency department setting [20]. Reliability of passive leg raising test is limited for spontaneously breathing patients or with intra-abdominal hypertension. The test may also be inconvenient in case of pain [18,19,21]. Hence, there is a need for additional means to guide fluid therapy in patients with sepsis.

There is evidence that bedside lung ultrasound can guide the fluid therapy. Positive net fluid balance correlates with extravascular lung water (EVLW) and is associated with higher mortality in patients with sepsis [5]. EVLW detection by the Fluid Administration Limited by Lung Sonography (FALLS)-protocol can be used to monitor acute circulatory failure based on the presence of B lines [22,23]. B lines are related to the thickening of interlobular septa, which is a pathological ultrasound sign [22,24,25]. Additionally, lung ultrasound is used to detect pulmonary edema with a sensitivity and specificity of 97% and 95%, respectively [22,25].

Observational studies suggest that the number of B lines correlates with the amount of EVLW. There is an inverse correlation between the number of B lines and oxygenation measured as the ratio of the partial pressure of arterial oxygen (PaO_2_) to the fraction of inspired oxygen (FiO_2_) [26]. The PaO_2_/FiO_2_ ratio is an integral part of the Sequential Organ Failure Assessment (SOFA) score, a score to assess and diagnose sepsis severity [14,27,28]. Elevated SOFA scores are associated with higher mortality in patients with sepsis [29]. However, lung ultrasound for guiding individualized fluid treatment in sepsis has never been tested in a randomized controlled setting.

We propose to limit the initial fluid volume in the treatment of patients with sepsis in the emergency department by detecting EVLW with lung ultrasound. This approach could enable physicians to better assess and meet individual fluid needs of patients with sepsis. This proposal may also lead to an improved therapy regimen for initial sepsis treatment that avoids administration of excess fluid volume, in turn, limits the consequent damage and decreases mortality in this high-risk group.

Therefore, we plan a randomized controlled trial with the primary objective to assess if fluid administration guided by bedside lung ultrasound can lead to an improved oxygenation (PaO_2_/FiO_2_) 48 hours after fluid administration than that in the current standard of care fluid administration in adult patients with sepsis in the emergency department.

Secondary objectives are to determine whether fluid administration guided by bedside lung ultrasound positively impacts the course of treatment after 15 mL/kg fluid at 0, 6, 12, 24, and 48 hours. These objectives will be evaluated by PaO_2_/FiO_2_ ratio, pulmonary outcomes (pulmonary edema, acute respiratory distress syndrome, or the need for invasive mechanical ventilation), the severity of sepsis (SOFA score), kidney function (mean creatinine level), the volume of administered fluid, and 30-day mortality.

## Methods

### Study Design

**Figure 1:**
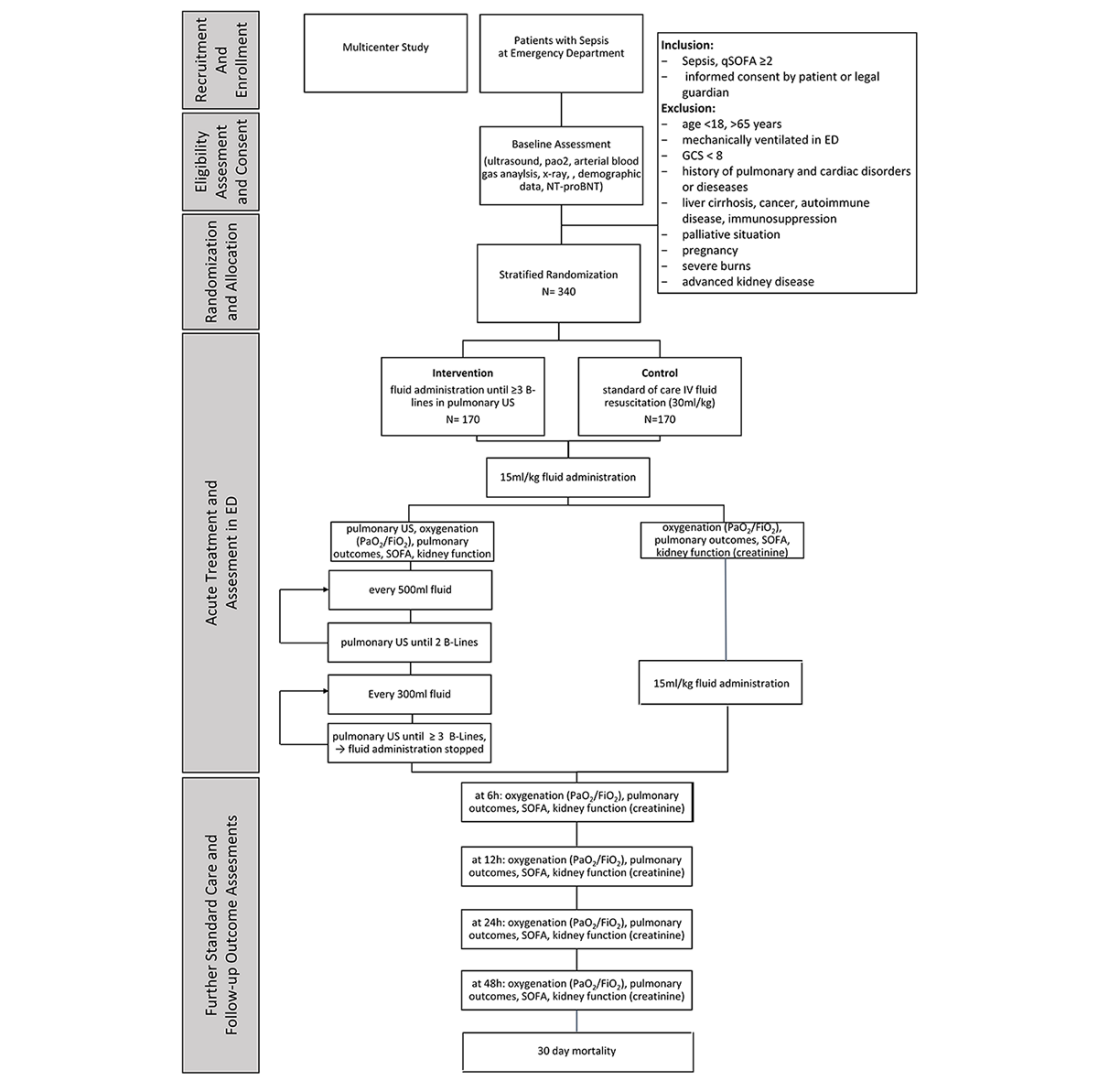
SPIRIT- and CONSORT-compliant flow diagram of study design. ED: emergency department; FiO2: fraction of inspired oxygen; GCS: Glasgow Coma Scale; IV: intravenous; PaO2: partial pressure of arterial oxygen; qSOFA: quick Sequential Organ Failure Assessment; SOFA: Sequential Organ Failure Assessment; US: ultrasound.

The trial (protocol version 1.1, April 2020; preprint version 1.0, April 2019 [30]) will be conducted as a prospective phase II multicenter, open-label with blinded endpoint assessment, parallel-group, randomized controlled trial. Study design will follow the Population, Intervention, Control, Outcome, and Time (PICOT) format (Multimedia Appendices 1 and 2). SPIRIT (Standard Protocol Items: Recommendations for Interventional Trials)- and CONSORT (Consolidated Standards of Reporting Trials)-compliant flow diagram is shown Figure 1.

Multimedia Appendices 2 and 3 respectively present SPIRIT-compliant flow diagram and checklist, respectively.

The schedule of enrollment, interventions and assessments is shown in Table 1.

**Table 1:** SPIRIT-compliant schedule of enrollment, interventions and assessments.

Study schedule sections		Study period								
		Enrollment	Allocation	Post-allocation						Prognosis
Time points		T1^a^	0 h	6 h	12 h	24 h	48 h	In-between	30 d	
**Enrollment**										
	Eligibility screening	X								
	Informed consent	X								
	Registration form	X								
	Baseline assessment (clinical features): sepsis with a qSOFA^b^ score≥2; low systolic blood pressure≤100 mmHg; respiratory rate≥22 breaths per minute; altered mentation with Glasgow coma scale score<15		X							
	Baseline assessment (laboratory parameters): ultrasound; PaO_2_/FiO_2_; arterial blood gas analysis; x-ray, NT-proBNP		X							
	Allocation		X							
**Interventions**										
	Intervention arm (fluid administration - 15mL/kg and ultrasound)		X	X	X	X	X			
	Placebo (standard-of-care - 30mL/kg)		X	X	X	X	X			
**Assessments**										
	Primary (PaO_2_/FiO_2_)^c^		X	X	X	X	X			
	Secondary: PaO_2_/FiO_2_/Pulmonary outcome/SOFA^d^ score/creatinine/volume		X	X	X	X	X			
	Prognosis (30-day mortality)								X	
	Documentation (amount of fluid)			X	X	X	X	X		

^a^T1: timepoint 1 before baseline; unknown units of time.

^b^qSOFA: quick Sequential Organ Failure Assessment.

^c^PaO_2_/FiO_2_: partial pressure of arterial oxygen/ fraction of inspired oxygen.

^d^SOFA: Sequential Organ Failure Assessment.

### Study Setting and Study Center Requirements

The study will be conducted as a multicenter study. Centers will be chosen and listed in the trial registration. Inclusion criteria will be as follows: patients with sepsis admission rate≥1000 patients/year; emergency department with necessary resources; personal and technical equipment (chest x-ray, point-of-care ultrasound devices, and access to blood analysis); intensive care unit (ICU); 24-hour availability of trained physicians; and academic hospital with an institutional review board conforming with the main center’s institutional review board). The participating centers will follow standardized written protocols for the evaluation and acute treatment of patients with suspected sepsis or septic shock.

To guarantee compliance with the protocol and application of the same technique, physicians responsible for ultrasound will participate in a practical workshop for lung ultrasound conducted by physicians from the main study center. These participant physicians must demonstrate their competency in lung ultrasounds (through 20 or more scans that will be validated by a main center radiologist or a point-of-care ultrasound–certified emergency department physician).

The physicians responsible for the treatment will have experience in the treatment of patients with sepsis following the hospital's standardized protocols and Sepsis-3 guidelines. Investigators following up on patients will be either physicians or trained nurses.

Adherence to the study requirements and standardized protocol will be ensured at the participating sites with periodic quality monitoring and training of the associated staff.

Further roles and details will be mentioned in the protocol registered after acquiring funding, ethical approval, and selecting participating centers [31].

### Recruitment and Adherence

Patients with suspected sepsis, that is, patients with a quick Sequential Organ Failure Assessment (qSOFA) score≥2 at the participating centers will be screened based on routine diagnostics and eligibility criteria and would consent to participate in the study. A low dropout rate is expected, due to short intervention time and fewer and brief follow-up periods. Nonetheless, participants will be informed about their right to withdraw from the study; and in the case of dropouts or withdrawal, the reasons will be documented. To achieve a sample size of 340 patients, a recruitment time of around 12-18 months is needed based on annually admission rate of 1000 patients and an expected recruitment rate of 5%-10% (50/1000-100/1000) per center. Multimedia Appendix 2 depicts the template for an attrition diagram [32]).

To ensure adherence, a standardized protocol and a checklist for the intervention will be provided to the participating centers and followed for each participant by the study investigators. The study investigators will document the patient's further treatment and monitor the treating department staff, to ensure that the protocol and necessary variables are documented. Patients or relatives will be reminded at the time of discharge from the hospital that they will be contacted for a follow-up.

### Eligibility Criteria

The eligibility criteria for patient recruitment is shown in Textbox 1.

**Inclusion and exclusion criteria for patient recruitment.**

Inclusion Criteria:

- Admitted into the emergency department.
- Age 18-65 years.
- Sepsis with a quick Sequential Organ Failure Assessment (qSOFA) score≥2 [3,14,28,33]
- Consent by the patient or legal guardian.

Exclusion Criteria:

- Mechanical ventilation at screening.
- Unconsciousness when admitted to emergency department (with Glasgow Coma Scale score<8)
- Preexisting pulmonary pathology as assessed by clinical symptoms or radiographic evidence in chest x-ray or by pulmonary bedside ultrasound (>2 B lines, a comet-tail artifact)
- History of pulmonary disorders (chronic obstructive pulmonary disease, asthma, parenchymal lung disease, or edema) or procedures.
- Preexisting cardiac pathology, disease, or dysfunction (ejection fraction<50, New York Health Association class>2) lung, or cardiac surgical procedures.
- History of liver cirrhosis, cancer, autoimmune disease, or immunosuppression.
- Patients under palliative care and patients facing imminent and inevitable death in the next 30 days due to causes other than sepsis
- Severe burns.
- Advanced kidney disease (chronic kidney disease stage 4 or above)
- Unstable medical conditions (eg, uncontrolled diabetes, uncompensated cardiac issues, heart failure, or chronic obstructive pulmonary disease).
- Pregnancy
- With other reasons or diseases needing a restricted fluid administration

### Randomization and Blinding

Randomization will be web-based across centers using simple randomization stratified for patient’s age and center, with 1:1 allocation. The sequence will be confidential and will be managed by a researcher independent of patient treatment. The treating physician will use the web-based randomization service—Viedoc (PCG Solutions AB). To increase the internal validity, possible confounders, such as pulmonary disorders, mechanical ventilation affecting imaging findings and bias results, kidney disease, and pre-existing cardiac disorders will be excluded by strictly implementing the eligibility criteria (Figure 1, Textbox 1, and Multimedia Appendix 2).

Patients and physicians will be unblinded to avoid endangering the patient's health and life in the situation of an emergency. However other study investigators collecting data, drawing blood samples for the analysis of PaO_2_/FiO_2_ and other parameters, and documenting, entering, or analyzing data will be blinded. Allocation concealment will be maintained until all data is collected and analyzed for the blinded personnel. The treating physician and patients will be instructed not to disclose information to the independent assessor and the data analyst. All breaches of blinding will need to be reported and documented.

### Intervention

**Figure 2:**
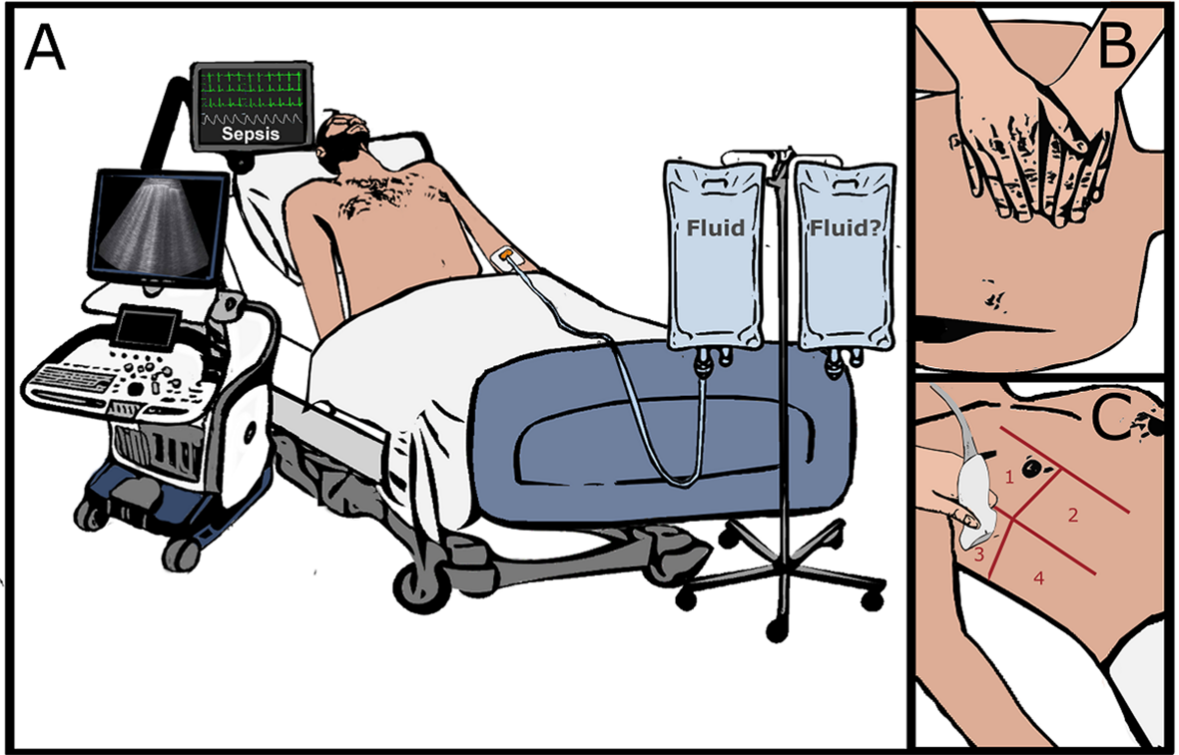
Bedside Lung Ultrasound in Emergency (BLUE) protocol.

Trained physicians will perform lung ultrasound following the Bedside Lung Ultrasound in Emergency (BLUE) protocol technique [34]—an ultrasound protocol to identify points at the thoracic cage of patients using both hands of the investigator (Figure 2)—to identify pulmonary edema by the appearance of B lines [26].

Panel A shows a patient with sepsis admitted to the emergency department. Patient will be surveyed for eligibility and will accordingly receive either fluid limited by lung ultrasound or 30 mL/kg (ie, the control group). Ultrasound will be performed as per the BLUE protocol. Panel B shows how to identify points on the thoracic cage of a patient through morphological examination using both hands: little finger of the upper hand is just below the clavicle, fingertips at middle line, and the lower hand below the upper hand. The upper BLUE-point is at the middle of the upper hand, and the lower BLUE-point is at the middle of the lower palm, creating 4 points in both hemithoraxes. At these points (1-4, as shown in panel C), ultrasound will be conducted and fluid will be given until 3 B lines appear in the ultrasound. BLUE protocol defines B lines as those vertical and echogenic narrow-based lines that widen progressively as they pass to the other end of the image [25,34].

A phased array (1-5 MHz) transducer will be used. The images will be acquired while the patient is in a supine position, saved, and coded for further quality control and assessing inter-rater reliability. A baseline lung-ultrasound scan to assess the extent of B lines (Figure 2) will be performed. Subsequently, the resuscitation target in the intervention arm will be determined or limited by lung ultrasound as follows (Figure 1). Repeated ultrasound scan will be performed after the initial 15 mL/kg IV fluid bolus in the intervention arm and then after every 500 mL of fluid administration. If 2 B lines appear, scans will be performed after every additional 300 mL of fluid in the intervention arm. When 3 or more B lines are present bilaterally in more than 2 areas, further fluid administration will be stopped. Therefore, fluid resuscitation target for the intervention group is to administer volume until the appearance of 3 B lines (Figure1). If a further hemodynamic compromise is detected in the intervention arm following IV fluid discontinuation, vasopressors will be started or continued as per the Sepsis-3 guidelines (Figure 1).

The control arm will be assessed by a baseline lung ultrasound to document the absence of B lines and will then receive at least 30 mL/kg fluids as per the Sepsis-3 guidelines. After the initial bolus, the type and rate of IV fluid administration will be left to the discretion of the treating physician and the local emergency departments’ protocols. All concomitant care and interventions are permitted during the trial.

### Outcomes

The primary outcome is the oxygenation (measured as mean PaO_2_/FiO_2_) at 48 hours after IV fluid administration. We expect a higher mean of PaO_2_/FiO_2_ in the intervention arm, which will be considered beneficial. A lower PaO_2_/FiO_2_ ratio is a sign of oxygenation compromise, which can occur with excessive fluid administration and increased EVLW. We will use PaO_2_/FiO_2_ ratio because it is a reliable and commonly used index of oxygenation that is easy to obtain and clinically significant as a predictor for mortality in patients with sepsis [26,35]. Arterial blood samples will be obtained for PaO_2_ measurement. FiO_2_ will be estimated based on widely accepted approximated values for those patients not mechanically ventilated [36].

The secondary outcomes will be assessed at time points of 0, 6, 12, 24, and 48 hours and after 15mL/kg of fluid administration, and also at 30 days. We will then evaluate (1) means of PaO_2_/FiO_2_ ratio between groups at these time points, (2) the incidence of pulmonary outcomes (pulmonary edema, acute respiratory distress syndrome, or need for invasive mechanical ventilation), (3) mean SOFA score, (4) mean creatinine level (in milligram per deciliter), (5) mean amount of volume of administered fluid, and (6) proportion frequency of 30-day mortality. Additionally, the type of IV fluid will be documented (Table 1).

Pulmonary outcomes (namely, pulmonary edema) will be confirmed by physicians based on symptoms and signs (eg, shortness of breath after fluid resuscitation and crackles), laboratory parameters (raised NT-proBNP, adjusted for age [37]), and x-ray findings suggestive of pulmonary edema.

The outcomes will be measured and documented in data collection forms by a nurse from the participating center. These forms will be made available in the protocol. Reliability and validity of laboratory tests at different participating centers will be made available in the protocol.

### Ethical Considerations

The protocol, templates, consent forms, and other requested documents (local language and English versions) will be reviewed and approved by the institutional review board/ethical committee at each participating site for the scientific content and regulatory compliance. Any modifications to the protocol will require a formal amendment and approval by the concerned institutional review board/ethical committee, notification to other research centers, and information update of trial registration [31].

Research investigators and physicians will have an informed discussion about the study details with conscious patients and provide printed information. The study investigator will use clinical judgment to discern between patients competent and incompetent to make a decision regarding their participation. Research investigators will obtain written consent from patients willing to participate in the study and will take the responsibility to protect those patients and follow ethical standards. Since it is likely that these patients might be ill or incompetent of taking decision at the time of giving consent. In such a case, the patient and their legal representative will receive an explanation about study details. They will be asked to provide an informed consent after expressing an understanding of the study procedures. When the patient is incapable of making a decision and there is no legal representative, the patient’s next of kin will be asked (either in-person or by phone) to provide a no-objection form based on his or her understanding of the patient’s wishes. If a no-objection form is provided, the patient will be included in the trial procedure since the intervention and assessment do not involve additional invasive or risky procedures. These patients will be retrospectively consented for their data to be included in the study as is the practice in other protocols [38]. Without a consent/no-objection form, the patient will be excluded from our study and will receive the usual care. We will reconsider the informed consent procedure repeatedly during the study in order to respect patients’ rights to withdraw from the study at any time. Strict data monitoring guidelines will be followed (Multimedia Appendix 4 Data Monitoring).

### Sample Size Justification

The sample size considerations were based on the PaO_2_/FiO_2_ differences between groups obtained from comparable prospective studies [26,39,40], where EVLW indices were used as a predictor for mortality. A PaO_2_/FiO_2_ ratio difference (delta) of 48 mm Hg was detected between the groups of survivors (mean 150, SD 81) and nonsurvivors (mean 198, SD78). Based on these studies, a delta of 25 mm Hg between the groups was considered clinically meaningful and feasible for our study. Interim analysis for safety reasons will be performed after 50% (170/ 340) of patient recruitment. An independent blinded statistician would perform the interim analysis and report to the data safety monitoring committee, as only safety is the monitored outcome no alpha level modification is needed (Multimedia Appendix 4 Interim analysis and safety).

The sample size was calculated (*α*=.05; ß=.80) with a potential dropout rate of 5% (17/340), resulting in n=170 patients per arm in a total of N=340 patients (Figure 1).

### Statistical Analysis

The primary outcome will be PaO_2_/FiO_2_, a continuous variable. Mean (SD) will be reported after testing for normality, and then will be analyzed via *t* test. For secondary outcomes, a repeated measure ANOVA and *t* test will be used for analysis of continuous data. Categorical data will be reported using proportions and will be analyzed using chi-square test.

All analysis will be conducted according to the principle of intention-to-treat, and multiple imputation technique will be used to account for missing data. A subgroup analysis will be performed based on randomization of age, site, and severity of sepsis. An intention-to-treat analysis approach will be used in this study (Multimedia Appendix 4 Missing Data).

### Limitations and Contingency Planning

Our study might lead to promising results but is not without limitations. The use of surrogate markers can be seen as a limitation. Using a surrogate marker to ensure study feasibility is universal in phase 2 trials. We will plan to study hard clinical outcomes in the following phase III trial. The chosen surrogate variable of PaO_2_/FiO_2_ ratio is a central component of the SOFA score [27,41]. Higher SOFA scores are associated with higher mortality [29]. Lower PaO_2_/FiO_2_ ratio is associated with adverse outcomes in patients with sepsis [41]. Additionally, the SOFA score will be assessed to evaluate changes in the patient’s status over time since it is a core component of Sepsis-3 guidelines [14,28] and has predictive value [42]. It represents a valuable additional surrogate variable. These surrogate variables will be supplemented by other variables essential for evaluating the progress of sepsis.

We have strict inclusion and exclusion criteria in order to enhance the detectability of differences between groups and show the efficacy of the intervention if present. However, this may limit the external validity of our results and slow down the recruitment process. (Multimedia Appendix 4 Contingency Planning).

Ultrasound is an operator-dependent intervention. To standardize the ultrasound technique and interpretation, trained physicians who prove competent will perform the ultrasound. Kappa statistics will be used to assess inter-rater reliability.

## Results

The study funding and ethical approval are being acquired, and the participating centers are being selected. The protocol will be registered with the intended registry name “Fluid administration in Emergency Room limited by Lung Ultrasound (FERLU) in patients with sepsis [31]: a phase II multicenter randomized controlled trial.” The protocol will follow the SPIRIT checklist (Multimedia Appendix 3) [43] and include all items from the World Health Organization Trial Registration Data Set. Details of sponsorship, complete protocol, and model consent forms will be provided. Changes will be regularly updated. The study is planned to be performed from 2021 to 2022, with enrollment starting in 2021. First results are expected in 2022. Based on the results of this study, independent recommendations will be made for potential further clinical trials and their designs. The results will be disseminated at international meetings in the fields of emergency medicine and intensive care and published in a peer-reviewed journal. The study will follow the authorship criteria of the International Committee of Medical Journal Editors for all publications.

## Discussion

The protocol and subsequent results can be the basis for an improved and individualized therapy regimen for initial sepsis treatment, which avoids damage resulting from excess fluid. It will be a further step toward new guidelines on tailored therapy approach for fluid administration in the management of sepsis.

Sepsis is one of the main reasons for ICU admissions, and 6%-30% of all ICU patients are assumed to suffer from sepsis [44]. The disease is associated with a high mortality and a considerable cost burden [45-47]. The adequate initial volume application is essential in the initial resuscitation of sepsis [8-11], and it affects patients’ outcome and mortality risk [4-6]. There is a need for individualized fluid resuscitation of patients with sepsis and septic shock at emergency departments [8-11]. Therefore, there is a need for reliable, fast, and easily applicable screening tools and protocols to individualize the fluid amount for every patient. This approach could be also embedded as a telemedicine expert consultation to save resources [48]. Lung ultrasound following the FALLS-protocol could be a feasible and aiding approach [22,24].

Lung ultrasound as per the FALLS-protocol is not well validated yet. Therefore, our proposed trial, as a first randomized clinical trial, will lay the ground for developing and validating the bedside lung ultrasound protocol for detecting EVLW as a tool guiding early treatment in the emergency department and help to validate the FALLS-protocol usage in emergency departments.

In this phase II multicenter parallel-group superiority trial, we will guide the fluids using bedside lung ultrasound in patients with sepsis evaluated by B lines as a marker for EVLW. Based on previous literature, it is hypothesized that patients receiving individually-adapted fluid therapy limited by lung ultrasound have better oxygenation leading to a better outcome and lower risk of mortality. With positive results, the study would proceed to a phase III with more liberal inclusion and exclusion criteria mimicking a real-life scenario. FERLU trial will lead to a deeper understanding of the fluid response in sepsis. The results of this study could help to decrease the mortality in this high-risk group of patients with sepsis by providing physicians an additional tool applicable in the emergency.

## Appendix

Multimedia Appendix 1 FERLU research question in PICOT format.

Multimedia Appendix 2 Template for an attrition diagram.

Multimedia Appendix 3 SPIRIT-compliant checklist for the FERLU trial.

Multimedia Appendix 4 Supplemental information.

## Abbreviations

ARISE: Australasian Resuscitation in Sepsis Evaluation
BLUE: Bedside Lung Ultrasound in Emergency
CONSORT: Consolidated Standards of Reporting Trials
EVLW: extra vascular lung water
FALLS: Fluid Administration Limited by Lung Sonography
FERLU: Fluid administration in Emergency Room limited by Lung Ultrasound FiO2: fraction of inspired oxygen
ICU: intensive care unit
IV: intravenous
PaO2: partial pressure of arterial oxygen
PICOT: Population, Intervention, Control, Outcome, and Time
ProCESS: Protocolized Care for Early Septic Shock
ProMISe: Protocolised Management in Sepsis
qSOFA: quick Sequential Organ Failure Assessment
SOFA: Sequential Organ Failure Assessment
SPIRIT: Standard Protocol Items: Recommendations for Interventional Trials

## References

[1] Fleischmann C, Scherag A, Adhikari NKJ, Hartog CS, Tsaganos T, Schlattmann P, Angus DC, Reinhart K, International FOACT (2016). Assessment of Global Incidence and Mortality of Hospital-treated Sepsis. Current Estimates and Limitations. Am J Respir Crit Care Med.

[2] Dellinger RP, Carlet JM, Masur H, Gerlach H, Calandra T, Cohen J, Gea-Banacloche J, Keh D, Marshall JC, Parker MM, Ramsay G, Zimmerman JL, Vincent J, Levy MM (2004). Surviving Sepsis Campaign guidelines for management of severe sepsis and septic shock. Intensive Care Med.

[3] Levy MM, Evans LE, Rhodes A (2018). The Surviving Sepsis Campaign Bundle: 2018 Update. Crit Care Med.

[4] Andrews B, Semler MW, Muchemwa L, Kelly P, Lakhi S, Heimburger DC, Mabula C, Bwalya M, Bernard GR (2017). Effect of an Early Resuscitation Protocol on In-hospital Mortality Among Adults With Sepsis and Hypotension: A Randomized Clinical Trial. JAMA.

[5] Boyd JH, Forbes J, Nakada T, Walley KR, Russell JA (2011). Fluid resuscitation in septic shock: a positive fluid balance and elevated central venous pressure are associated with increased mortality. Crit Care Med.

[6] Sirvent J, Ferri C, Baró A, Murcia C, Lorencio C (2015). Fluid balance in sepsis and septic shock as a determining factor of mortality. Am J Emerg Med.

[7] Rivers E, Nguyen B, Havstad S, Ressler J, Muzzin A, Knoblich B, Peterson E, Tomlanovich M, Early Goal-Directed Therapy Collaborative Group (2001). Early goal-directed therapy in the treatment of severe sepsis and septic shock. N Engl J Med.

[8] Byrne L, Van Haren F (2017). Fluid resuscitation in human sepsis: Time to rewrite history?. Ann Intensive Care.

[9] Mouncey PR, Osborn TM, Power GS, Harrison DA, Sadique MZ, Grieve RD, Jahan R, Tan JCK, Harvey SE, Bell D, Bion JF, Coats TJ, Singer M, Young JD, Rowan KM (2015). Protocolised Management In Sepsis (ProMISe): a multicentre randomised controlled trial of the clinical effectiveness and cost-effectiveness of early, goal-directed, protocolised resuscitation for emerging septic shock. Health Technol Assess.

[10] Peake Sandra L, Delaney Anthony, Bailey Michael, Bellomo Rinaldo, Cameron Peter A, Cooper D James, Higgins Alisa M, Holdgate Anna, Howe Belinda D, Webb Steven A R, Williams Patricia, ARISE Investigators, ANZICS Clinical Trials Group (2014). Goal-directed resuscitation for patients with early septic shock. N Engl J Med.

[11] ProCESS Investigators T, Yealy Donald M, Kellum John A, Huang David T, Barnato Amber E, Weissfeld Lisa A, Pike Francis, Terndrup Thomas, Wang Henry E, Hou Peter C, LoVecchio Frank, Filbin Michael R, Shapiro Nathan I, Angus Derek C (2014). A randomized trial of protocol-based care for early septic shock. N Engl J Med.

[12] Carneiro AH, Póvoa P, Gomes JA (2017). Dear Sepsis-3, we are sorry to say that we don't like you. Rev Bras Ter Intensiva.

[13] Sartelli M, Kluger Y, Ansaloni L, Hardcastle TC, Rello J, Watkins RR, Bassetti M, Giamarellou E, Coccolini F, Abu-Zidan FM, Adesunkanmi AK, Augustin G, Baiocchi GL, Bala M, Baraket O, Beltran MA, Jusoh AC, Demetrashvili Z, De Simone B, de Souza HP, Cui Y, Davies RJ, Dhingra S, Diaz JJ, Di Saverio S, Dogjani A, Elmangory MM, Enani MA, Ferrada P, Fraga GP, Frattima S, Ghnnam W, Gomes CA, Kanj SS, Karamarkovic A, Kenig J, Khamis F, Khokha V, Koike K, Kok KYY, Isik A, Labricciosa FM, Latifi R, Lee JG, Litvin A, Machain GM, Manzano-Nunez R, Major P, Marwah S, McFarlane M, Memish ZA, Mesina C, Moore EE, Moore FA, Naidoo N, Negoi I, Ofori-Asenso R, Olaoye I, Ordoñez CA, Ouadii M, Paolillo C, Picetti E, Pintar T, Ponce-de-Leon A, Pupelis G, Reis T, Sakakushev B, Kafil HS, Sato N, Shah JN, Siribumrungwong B, Talving P, Tranà C, Ulrych J, Yuan K, Catena F (2018). Raising concerns about the Sepsis-3 definitions. World J Emerg Surg.

[14] Seymour CW, Liu VX, Iwashyna TJ, Brunkhorst FM, Rea TD, Scherag A, Rubenfeld G, Kahn JM, Shankar-Hari M, Singer M, Deutschman CS, Escobar GJ, Angus DC (2016). Assessment of Clinical Criteria for Sepsis: For the Third International Consensus Definitions for Sepsis and Septic Shock (Sepsis-3). JAMA.

[15] Cherpanath TGV, Geerts BF, Lagrand WK, Schultz MJ, Groeneveld ABJ (2013). Basic concepts of fluid responsiveness. Neth Heart J.

[16] Sanfilippo F, Corredor C, Fletcher N, Landesberg G, Benedetto U, Foex P, Cecconi M (2015). Diastolic dysfunction and mortality in septic patients: a systematic review and meta-analysis. Intensive Care Med.

[17] Cavallaro F, Sandroni C, Marano C, La Torre G, Mannocci A, De Waure C, Bello G, Maviglia R, Antonelli M (2010). Diagnostic accuracy of passive leg raising for prediction of fluid responsiveness in adults: systematic review and meta-analysis of clinical studies. Intensive Care Med.

[18] Teboul J, Monnet X (2008). Prediction of volume responsiveness in critically ill patients with spontaneous breathing activity. Curr Opin Crit Care.

[19] Monnet X, Marik P, Teboul J (2016). Passive leg raising for predicting fluid responsiveness: a systematic review and meta-analysis. Intensive Care Med.

[20] Porhomayon J, Zadeii G, Congello S, Nader ND (2012). Applications of minimally invasive cardiac output monitors. Int J Emerg Med.

[21] Teboul J, Saugel B, Cecconi M, De Backer D, Hofer CK, Monnet X, Perel A, Pinsky MR, Reuter DA, Rhodes A, Squara P, Vincent J, Scheeren TW (2016). Less invasive hemodynamic monitoring in critically ill patients. Intensive Care Med.

[22] Lichtenstein D (2013). FALLS-protocol: lung ultrasound in hemodynamic assessment of shock. Heart Lung Vessel.

[23] Lichtenstein DA (2015). BLUE-protocol and FALLS-protocol: two applications of lung ultrasound in the critically ill. Chest.

[24] Miller A (2016). Practical approach to lung ultrasound. BJA Education.

[25] Lichtenstein D, Mezière G (1998). A lung ultrasound sign allowing bedside distinction between pulmonary edema and COPD: the comet-tail artifact. Intensive Care Med.

[26] Theerawit P, Touman N, Sutherasan Y, Kiatboonsri S (2014). Transthoracic ultrasound assessment of B-lines for identifying the increment of extravascular lung water in shock patients requiring fluid resuscitation. Indian J Crit Care Med.

[27] Vincent JL, Moreno R, Takala J, Willatts S, De Mendonça A, Bruining H, Reinhart CK, Suter PM, Thijs LG (1996). The SOFA (Sepsis-related Organ Failure Assessment) score to describe organ dysfunction/failure. On behalf of the Working Group on Sepsis-Related Problems of the European Society of Intensive Care Medicine. Intensive Care Med.

[28] Singer M, Deutschman CS, Seymour CW, Shankar-Hari M, Annane D, Bauer M, Bellomo R, Bernard GR, Chiche J, Coopersmith CM, Hotchkiss RS, Levy MM, Marshall JC, Martin GS, Opal SM, Rubenfeld GD, van DPT, Vincent J, Angus DC (2016). The Third International Consensus Definitions for Sepsis and Septic Shock (Sepsis-3). JAMA.

[29] Ferreira FL, Bota DP, Bross A, Mélot C, Vincent JL (2001). Serial evaluation of the SOFA score to predict outcome in critically ill patients. JAMA.

[30] Mohamed MFH, Malewicz N, Zehry H, Hussain D, Barouh JA, Cançado Adriana V, Silva Jeancarllo De Sousa, Suwileh Salah, Carvajal Jose Retamal (2020). Protocol for Fluid administration in Emergency Departments limited by Lung Ultrasound (FERLU) in patients with sepsis: a multi-center randomized, controlled, phase II trial. JMIR Preprints.

[31] ClinicalTrials.gov.

[32] Eysenbach G, CONSORT-EHEALTH Group (2011). CONSORT-EHEALTH: improving and standardizing evaluation reports of Web-based and mobile health interventions. J Med Internet Res.

[33] Vincent JL, de Mendonça A, Cantraine F, Moreno R, Takala J, Suter PM, Sprung CL, Colardyn F, Blecher S (1998). Use of the SOFA score to assess the incidence of organ dysfunction/failure in intensive care units: results of a multicenter, prospective study. Working group on sepsis-related problems of the European Society of Intensive Care Medicine. Crit Care Med.

[34] Lichtenstein DA, Mezière GA (2008). Relevance of lung ultrasound in the diagnosis of acute respiratory failure: the BLUE protocol. Chest.

[35] El-Khatib MF (2008). Oxygenation indexes and degrees of lung injury. Am J Respir Crit Care Med.

[36] Shapiro BA (1995). The history of pH and blood gas analysis. Respir Care Clin N Am.

[37] Januzzi JL, van Kimmenade R, Lainchbury J, Bayes-Genis A, Ordonez-Llanos J, Santalo-Bel M, Pinto YM, Richards M (2006). NT-proBNP testing for diagnosis and short-term prognosis in acute destabilized heart failure: an international pooled analysis of 1256 patients: the International Collaborative of NT-proBNP Study. Eur Heart J.

[38] Watkinson PJ, Barber VS, Young JD (2018). Outcome of Critically ill Patients Undergoing Mandatory Insulin Therapy Compared to Usual Care Insulin Therapy: Protocol for a Pilot Randomized Controlled Trial. JMIR Res Protoc.

[39] Caltabeloti F, Monsel A, Arbelot C, Brisson H, Lu Q, Gu W, Zhou G, Auler JOC, Rouby J (2014). Early fluid loading in acute respiratory distress syndrome with septic shock deteriorates lung aeration without impairing arterial oxygenation: a lung ultrasound observational study. Crit Care.

[40] Chung F, Lin S, Lin S, Lin H (2008). Impact of extravascular lung water index on outcomes of severe sepsis patients in a medical intensive care unit. Respir Med.

[41] Pandharipande PP, Shintani AK, Hagerman HE, St Jacques PJ, Rice TW, Sanders NW, Ware LB, Bernard GR, Ely EW (2009). Derivation and validation of Spo2/Fio2 ratio to impute for Pao2/Fio2 ratio in the respiratory component of the Sequential Organ Failure Assessment score. Crit Care Med.

[42] Minne L, Abu-Hanna A, de Jonge E (2008). Evaluation of SOFA-based models for predicting mortality in the ICU: A systematic review. Crit Care.

[43] Schulz KF, Grimes DA (2013). Get in the spirit with SPIRIT 2013: protocol content guideline for clinical trials. Contraception.

[44] Desautels T, Calvert J, Hoffman J, Jay M, Kerem Y, Shieh L, Shimabukuro D, Chettipally U, Feldman MD, Barton C, Wales DJ, Das R (2016). Prediction of Sepsis in the Intensive Care Unit With Minimal Electronic Health Record Data: A Machine Learning Approach. JMIR Med Inform.

[45] Avila AA, Kinberg EC, Sherwin NK, Taylor RD (2016). The Use of Fluids in Sepsis. Cureus.

[46] Bansal M, Farrugia A, Balboni S, Martin G (2013). Relative survival benefit and morbidity with fluids in severe sepsis - a network meta-analysis of alternative therapies. Curr Drug Saf.

[47] Joshi M, Ashrafian H, Arora S, Khan S, Cooke G, Darzi A (2019). Digital Alerting and Outcomes in Patients With Sepsis: Systematic Review and Meta-Analysis. J Med Internet Res.

[48] Deisz R, Rademacher S, Gilger K, Jegen R, Sauerzapfe B, Fitzner C, Stoppe C, Benstoem C, Marx G (2019). Additional Telemedicine Rounds as a Successful Performance-Improvement Strategy for Sepsis Management: Observational Multicenter Study. J Med Internet Res.

