# Supplementary figures and images for: Fluid Administration in Emergency Room Limited by Lung Ultrasound in Patients with Sepsis: Protocol for a Prospective Phase II Multicenter Randomized Controlled Trial
Source: JMIR Res Protoc. 2020 Aug 26;9(8):e15997. doi: 10.2196/15997 (PMC7481877; doi:10.2196/15997)

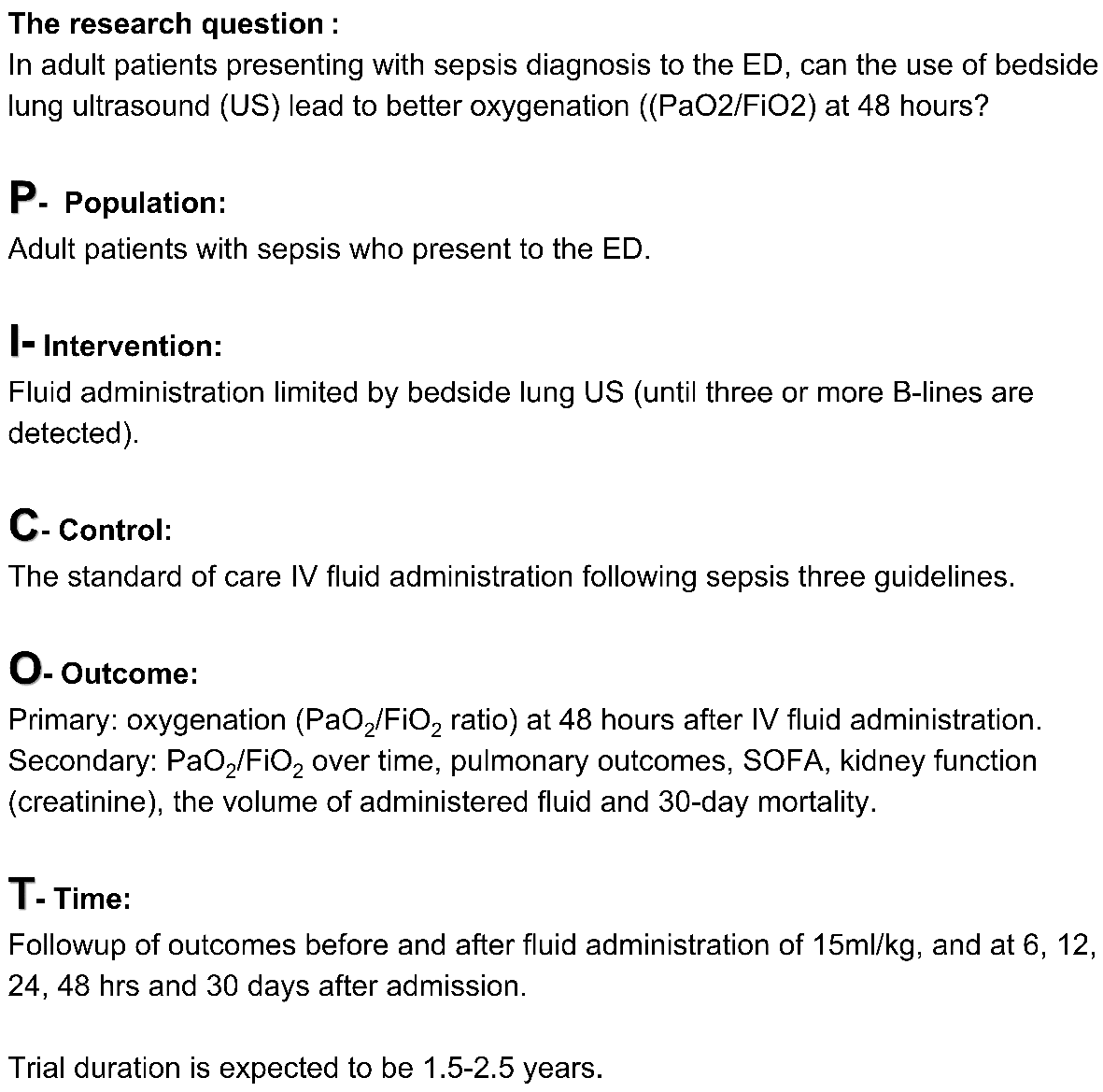

Supplement: Multimedia Appendix 1 [file resprot_v9i8e15997_app1.PNG]

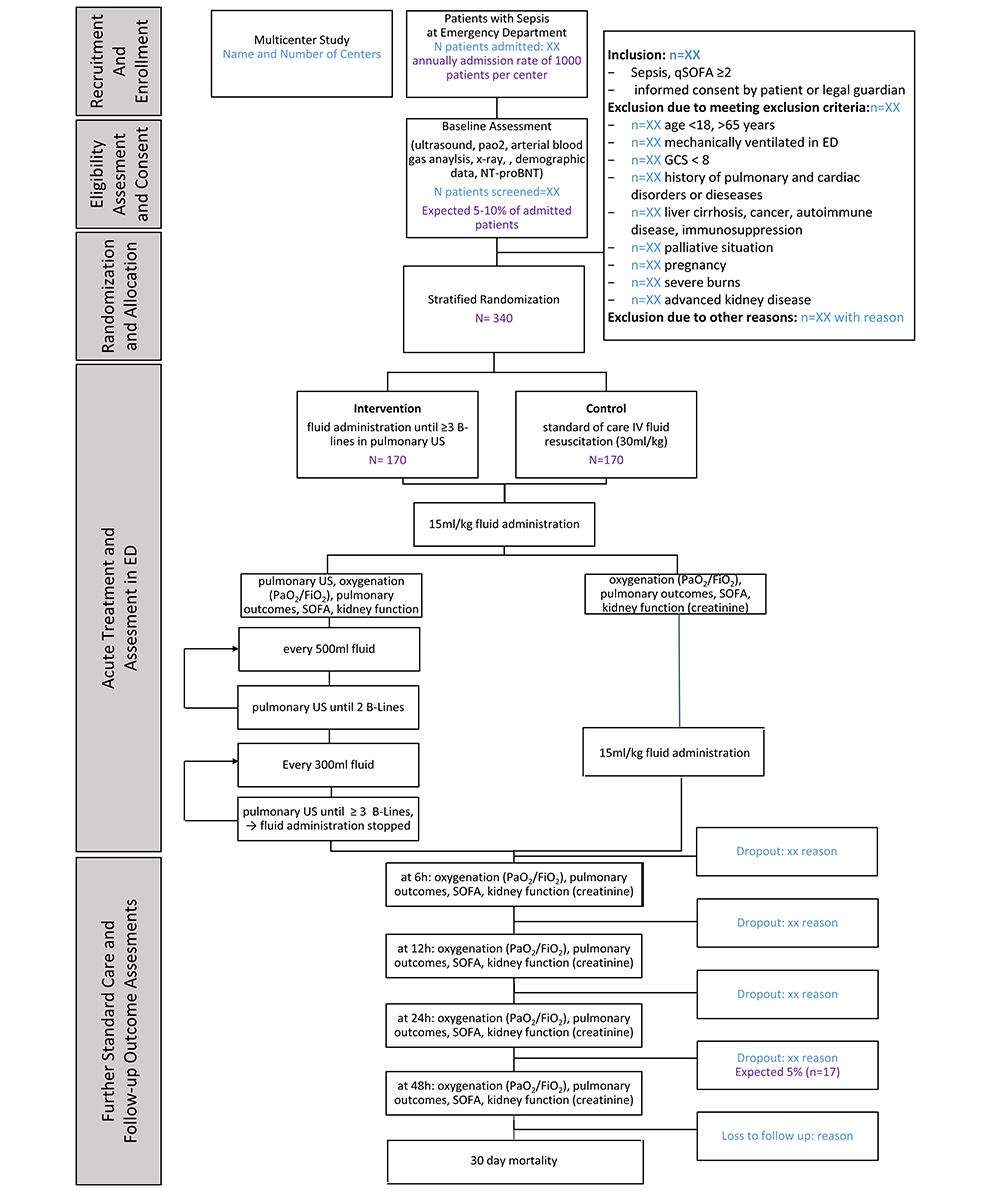

Supplement: Multimedia Appendix 2 [file resprot_v9i8e15997_app2.PNG]
