# Supplementary material for: Fluid Administration in Emergency Room Limited by Lung Ultrasound in Patients with Sepsis: Protocol for a Prospective Phase II Multicenter Randomized Controlled Trial
Source: JMIR Res Protoc. 2020 Aug 26;9(8):e15997. doi: 10.2196/15997 (PMC7481877; doi:10.2196/15997)
Supplement: Multimedia Appendix 4 [file resprot_v9i8e15997_app4.docx]

# Supplemental information

### Data Monitoring

Participants information will be de-identified, assigned and ID coded, the accompanying name will then be deleted for future data handling to ensure patients' confidentiality. All subjects' collected data from will be maintained in a secure and password protected database. Un-blinded study personnel will not have access to this database. Besides, all records that contain names or other personal identifiers, (i.e., IRB approved questionnaires in subject folders) will be stored separately from study records identified by a code number. Moreover, the data dispersed to project team members will be blinded of any identifying participant information. All authors and participating and principal investigators will have to disclose any potential conflict of interest and need to update a provided COI checklist regularly.

### Interim analysis and safety

The data safety monitoring committee (DSMC) is responsible for evaluating the interim analysis results, overall conduct of the clinical trial and protocol adherence to ensure valid results, enriching clinical knowledge and practice. Interim-analysis for safety reasons will be performed after 50% of patients’ recruitment. An independent blinded statistician would perform the interim analysis, and report to DSMC, as only safety is the monitored outcome no alpha level modification is needed.

In case of severe adverse pulmonary outcomes, kidney impairment or death, the event will be reported possible relation to the study intervention and assessed. The DSMC will be advisory to the Trial Steering Committee (TSC). TSC will be responsible for promptly reviewing the DSMC recommendations, to decide whether to continue or terminate the trial and to determine whether amendments to the protocol or changes in study conduct are required.

DSMC membership will be restricted to individuals free of relevant significant conflicts of interest related to the conduct, outcome, or impact of the study.  All DSMC members must disclose all possible conflicts of interest in writing before inclusion as DSMC members. Newly arising activities potentially posing a COI must be updated and reported. DSMC membership will be assigned generally for the duration of the clinical trial. If any member leaves the DSMC during the trial, the sponsor, in consultation with the TSC and investigators will promptly appoint a replacement.

The trial termination rules, meeting intervals as well as the structure, independence, and members of the DSMC of this study will be stated in the final protocols approved by the local IRBS and updated in the clinical trial registration and will include the organizational relationship between DSMC and study stakeholders. Information about auditing, role and access of study sponsor and funders, composition and role of coordinating center, steering committee will be provided in the final protocols approved by the local IRBs and updated in the clinical trial registration. Further roles and details of the study sponsors and funders, composition, roles, responsibilities of the coordinating center and participating centers, list of countries and communities will be mentioned in the protocol registered after acquiring funding, ethical approval and selecting centers to participate [31].Patients and public have not been involved in devising the protocol.

### Missing Data

An intention to treat analysis approach will be used in this study. As the trial duration is short and the number of measurements of the PaO_2_/FiO_2_ is kept to the minimal possible, we are not expecting a significant number of missing data. If missing data are related to the primary outcome, then these will be most likely related to the nature of the intervention where some patients may refuse repeated blood gas analysis or due to dropping out or withdrawal from the study. In these scenarios, the missing data will be considered as missing at random and multiple imputation methods will be implemented to generate the missing values. In the case of any dropouts or withdrawals from the study, the reasons will be studied and documented. Any other missing data will be assessed by sensitivity analysis to check their impact on the study outcomes. The Data Monitoring Committee (DMC) will take the responsibility to monitor for any missing data to ensure that actions are taken timely.

Contingency Planning

Our study might lead to promising results but is not without limitations. Therefore contingency planning is essential to provide a solution and foresee strategies and alternative processes. The use of surrogate markers can be seen as a limitation. Using a surrogate marker to ensure study feasibility is universal in phase 2 trials, we will plan to study hard clinical outcomes in the following phase III trial. The chosen surrogate variable of PaO_2_/FiO_2_ ratio is a central component of the SOFA score [27, 41]. The higher the SOFA score, the higher the mortality [29]. Lower PaO_2_/FiO_2_ ratio is associated with adverse outcomes in patient with sepsis [41], Additionally, the SOFA score will be assessed to evaluate changes in patient status over time, since it is a core component of Sepsis 3 guidelines [14, 28] and has predictive value [42], it represents a valuable additional surrogate variable. These surrogate variables will be supplemented by other variables essential for evaluating the progress of sepsis.

We have strict inclusion and exclusion criteria in order to enhance the signal and show the efficacy of the intervention if present, but this may limit the external validity of our results, moreover, slows the recruitment process, that is why we planned multicenter study to enhance the recruitment, and we allocated a reasonable study timeframe for study completion. To complete the study in the timeframe the participating centers will be chosen based on a minimum annual sepsis admission rate. Moreover, posters & leaflets will be distributed in the EDs of the participating centers to increase awareness of our study in the population and enhance recruitment.

The high frequency of ultrasound monitoring is another limitation. We planned frequent US monitoring to diagnose EVLW promptly, in phase III trial we will adopt a simpler less frequent protocol that may suit the busy emergency setting better, yet helps to improve the patients care by tailoring fluid therapy. To solve this concern, an independent investigator will be present also to perform the ultrasound if needed.

Ultrasound is an operator-dependent intervention, to standardize the US technique and interpretation, trained physicians who prove competent will perform the ultrasound, and Kappa statistics will be used to assess inter-rater reliability.

Diverse measures as discussed in paragraph 2.3 recruitment and adherence will be followed to ensure following the protocol.
